# Organic Microanalysis of Submicrogram Samples

**DOI:** 10.6028/jres.093.032

**Published:** 1988-06-01

**Authors:** Douglas B. Hooker, Jack DeZwaan

**Affiliations:** The Upjohn Company, Kalamazoo, MI 49001

## 1. Introduction

Information on the elemental composition of materials can be of great value in a variety of research problems. Obtaining this information is a problem in cases where only a very small amount (micrograms) of material is available since conventional microanalytical techniques require much larger (milligram) samples for each elemental determination. In these situations, a method which would determine all the elements present using a single, submicrogram sample would be useful.

Atomic emission generated from samples which have been introduced into a microwave plasma (MIP) has been investigated extensively as a means of element specific detection and as a means of elemental ratio determination [1,2]. Microwave plasmas generated in a helium carrier are especially useful because high levels of excited atomic states of several interesting nonmetals such as carbon, nitrogen, phosphorous, sulfur and the halogens can be generated [3,4]. Simultaneous measurement of emission from the desired elements provides the potential for multielemental determination using a single submicrogram sample.

Although gas phase procedures for introducing samples into microwave plasmas have been the most reliable, they cannot be used with most solid samples. Electro-thermal procedures have also been reported for some sample types [5,6]. The more general method of sample introduction for nonvolatile samples reported here uses a fine quartz filament to deliver a sample to a low pressure microwave plasma. This procedure delivers the sample to the plasma intact, producing sharp responses of around a second in duration at all emission wavelengths. A series of samples containing C, S, F, and Cl are used to evaluate the performance of this technique in determining elemental ratios in solid, nonvolatile samples and in predicting empirical formulas with complementary mass spectral data.

## 2. Experimental

The data were generated on a commercial instrument, the MPD 850 (Applied Chromatography Systems, Lutton, Bedfordshire, U.K.). This instrument was designed as a gas chromatographic detector and was modified for solid sample introduction. The MIP was produced in a 1/4-wave Evanson type cavity using chromatographic grade helium at ~ 10 torr, containing about 0.2% oxygen to prevent carbon buildup on walls of the quartz tube confining the plasma. Forward microwave power of 100 watts at 2.45 GHz was used with minimal reflected power. The grating produced a reciprocal dispersion of 1.39 nm/mm (first order).

Elemental emission was detected simultaneously at the wavelengths shown in table 1. The photocurrents were amplified and digitized (120 Hz) and stored on a Harris H1000 computer.

**Figure 1:**
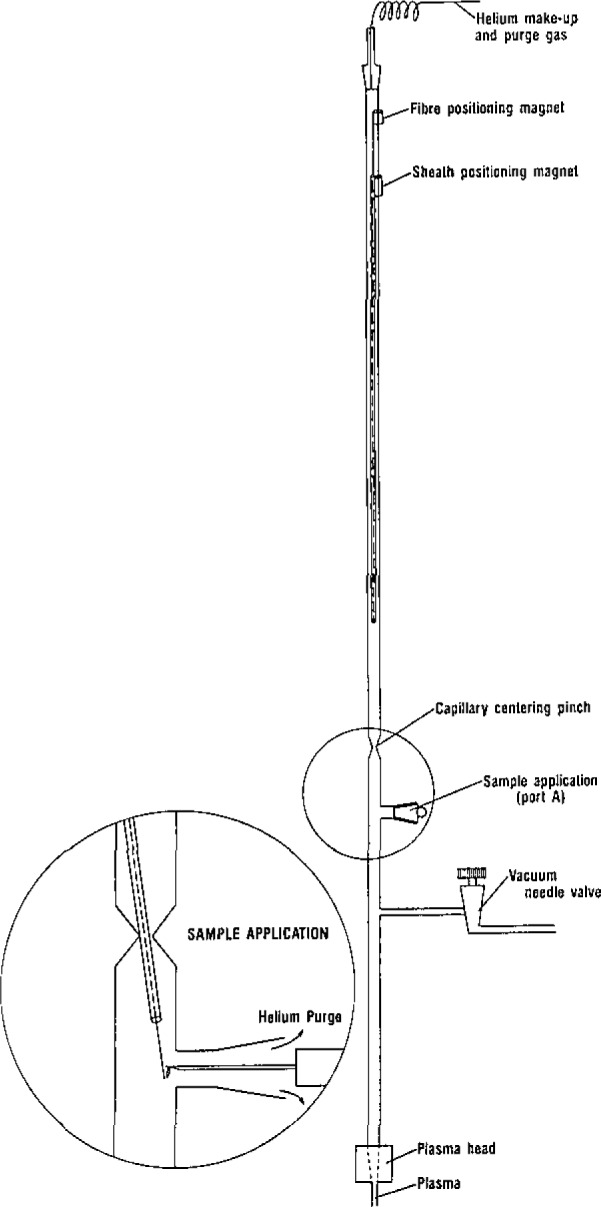
Device used to deliver solid samples to the plasma. Sample is loaded onto the quartz filament at port A using a Hamilton syringe as shown in the insert.

The sample delivery device, illustrated in figure 1, consists of a quartz Filament (0.0776–0.102 mm diameter) inside a hollow-fused silica guide (OD=0.42 mm and ID=0.32 mm). This assembly was contained within the enclosed vacuum manifold. Individual magnets were attached to both the filament and the guide tube which allowed them to be moved in tandem or individually with magnets on the outside of the manifold.

Samples were loaded from chloroform solution directly onto the quartz fiber using a 10 μL Hamilton syringe at Port A. The solvent was allowed to evaporate before analysis. Complete solvent removal was established by the absence of carbon or chlorine atomic emission after the plasma was triggered. After being loaded, the fiber was drawn up into the guide tube and the assembly was lowered in tandem to a point just above the plasma. The fiber was then lowered directly into the upper portion of the plasma. After sample vaporization the fiber could be raised back into the guide tube and the assembly raised in the manifold for reloading.

## 3. Results and Discussion

**Figure 2:**
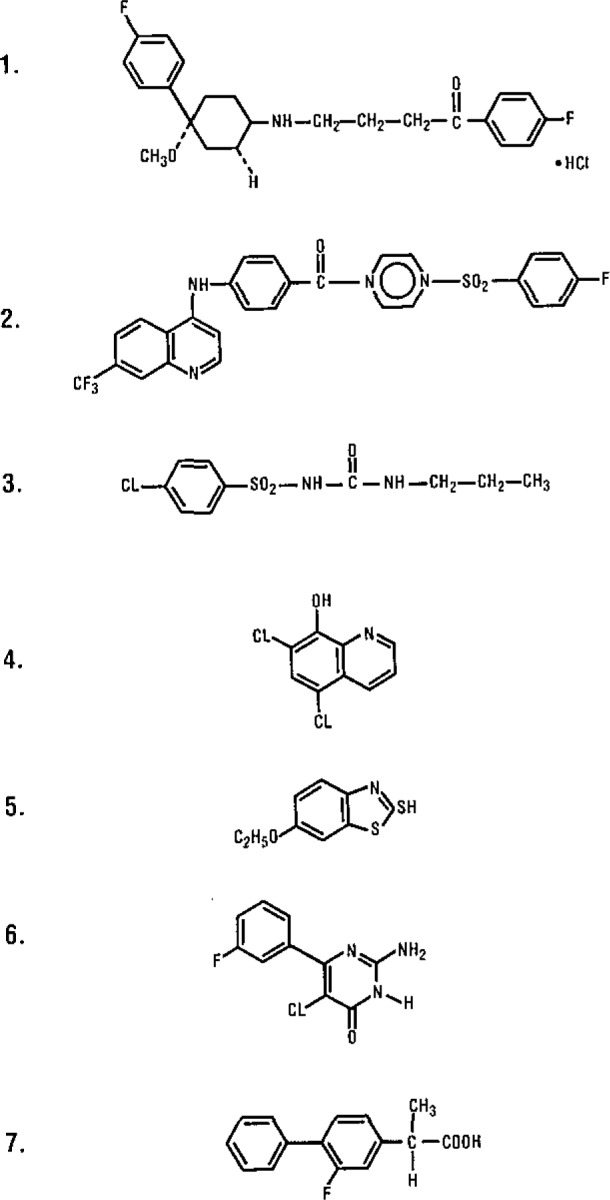
Structures of the nonvolatile samples studied.

**Figure 3:**
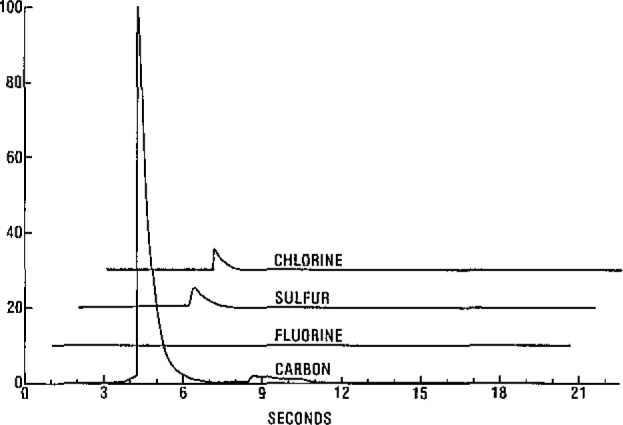
Typical responses obtained for a 500 ng sample.

The structures of the samples used are given in figure 2. The responses shown in figure 3 are typical of those obtained using sample sizes of about 500 nanograms. For illustration they have been offset and normalized so that the largest signal is full scale. The responses are all sharp with a half-width of under a second and a high signal to noise ratio for all elements. Within seconds of the initial analysis, the quartz fiber was reinserted into the plasma. No observable responses were observed for any of the samples following this procedure indicating that the sample was completely transferred to the plasma on the initial insertion.

The elemental responses were quantitated by peak integrations and these responses were used in eq (1) to give the data shown in table 2.

$$X_{\text{sam}}=\frac{c_{\text{std}}}{x_{\text{std}}}\cdot \frac{X_{\text{std}}}{C_{\text{std}}}\cdot \frac{x_{\text{sam}}}{c_{\text{sam}}}C_{\text{sam}},$$ (1)

where *c* and *x* are the integrals obtained for carbon (*c*) and the heteroatom (*x*) and C and X are the number of carbons (C) and heteroatoms (X) appearing in the empirical formula.

Each of the responses in table 2 is the average of four independent sample runs, with at least two different quartz fibers and plasma tubes used for each compound. When the experimental values are rounded to the nearest whole number they agree with the value expected from the empirical formula.

While it is true that the value of C_sam_ in the above expression will not be known for actual samples, the measured mass of the material is often available from mass spectrometry. Knowing the exact mass allows information on possible empirical formulae to be generated especially if information on the heteroatoms present is available. This is illustrated in table 3 for the mass of 502.1226 derived from the empirical formula C_27_H_22_F_4_O_3_S. Using all possible values of carbon from tables 3 as C_sam_ in eq (1) along with the ratio of area responses obtained for compound 1 in figure 2, it is possible to determine the empirical formula of this sample.

While the unique selection of an empirical formula is not always possible, the number of choices is always greatly reduced using information on elemental ratios especially if the number of elements measured is increased.

## Figures and Tables

**Table 1:** Wavelengths monitored and the slit widths used to monitor atomic emission

Element	Wavelength (nm)	Slit width (μm)
Carbon	247.86	75
Chlorine	479.45	50
Sulfur	545.39	50
Fluorine	685.60	75

**Table 2:** The number of heteroatoms per molecule calculated from the experimentally determined elemental response ratios. Each result is the average of four separate determinations. The standard deviations are given in parentheses

Empirical Formula	F atoms	S atoms	Cl atoms
C_23_H_29_NO_2_F_2_Cl	2.4(0.11)	0.017	0.97 (0.25)
C_27_H_22_O_3_F_4_S^a^	4.0 (0.1)	0.91 (0.07)	0.1
C_10_H_13_N_2_O_3_SCl^b^	0.004	1.00 (0.03)	1.00 (0.11)
C_9_H_5_NOCl_2_	0.006	0.004	2.5 (0.4)
C_9_H_9_NOS_2_	0.001	2.3 (0.2)	0.03
C_10_H_7_NO_3_FCl	0.96(0.01)	0.004	1.1 (0.3)
C_15_H_13_O_2_F	0.83 (0.01)	0.007	0.02

a Used as calibration standard for F.

b Used as calibration standard for S and Cl.

**Table 3:** Empirical formulae within 5 millimass units of 502.1226 when heteroatoms are constrained to the ranges 0⩽F⩽5, 0⩽O⩽5 and 0⩽S⩽2

C	H	F	O	S
33	17	3	2	0
30	18	4	3	0
35	18	0	4	0
27	19	5	4	0
32	19	1	5	0
36	19	1	0	1
33	20	2	1	1
30	21	3	2	1
27	22	4	3	1
32	22	0	4	1
24	23	1	5	1
29	23	1	0	2
33	23	1	0	2
30	24	2	1	2
27	25	3	2	2
24	26	4	3	2
29	26	0	4	2
21	27	5	4	2
